# *IARS2* mutations lead to Leigh syndrome with a combined oxidative phosphorylation deficiency

**DOI:** 10.1186/s13023-024-03310-x

**Published:** 2024-08-21

**Authors:** Qiyu Dong, Xiaojie Yin, Shuanglong Fan, Sheng Zhong, Wenxin Yang, Keer Chen, Qian Wang, Xue Ma, Refiloe Laurentinah Mahlatsi, Yanling Yang, Jianxin Lyu, Hezhi Fang, Ya Wang

**Affiliations:** 1 Key Laboratory of Laboratory Medicine, Ministry of Education, Zhejiang Provincial Key Laboratory of Medical Genetics, School of Laboratory Medicine and Life Sciences, Wenzhou Medical University, Wenzhou, Zhejiang 325035 China; 2 Department of Pediatrics, Peking University First Hospital, Beijing, 100034 China; 3 Laboratory Medicine Center, Department of Clinical Laboratory, Zhejiang Provincial People’s Hospital, Affiliated People’s Hospital, Hangzhou Medical College, Hangzhou, Zhejiang 310053 China

**Keywords:** *IARS2*, Leigh syndrome, Mitochondrial disease, OXPHOS

## Abstract

**Background:**

Leigh syndrome (LS) is a common mitochondrial disease caused by mutations in both mitochondrial and nuclear genes. Isoleucyl-tRNA synthetase 2 (IARS2) encodes mitochondrial isoleucine-tRNA synthetase, and variants in *IARS2* have been reported to cause LS. However, the pathogenic mechanism of *IARS2* variants is still unclear.

**Methods:**

Two unrelated patients, a 4-year-old boy and a 5-year-old boy diagnosed with LS, were recruited, and detailed clinical data were collected. The DNA of the patients and their parents was isolated from the peripheral blood for the identification of pathogenic variants using next-generation sequencing and Sanger sequencing. The ClustalW program, allele frequency analysis databases (gnomAD and ExAc), and pathogenicity prediction databases (Clinvar, Mutation Taster and PolyPhen2) were used to predict the conservation and pathogenicity of the variants. The gene expression level, oxygen consumption rate (OCR), respiratory chain complex activity, cellular adenosine triphosphate (ATP) production, mitochondrial membrane potential (MMP) and mitochondrial reactive oxygen species (ROS) levels were measured in patient-derived lymphocytes and *IARS2*-knockdown HEK293T cells to evaluate the pathogenicity of the variants.

**Results:**

We reported 2 unrelated Chinese patients manifested with LS who carried biallelic *IARS2* variants (c.1_390del and c.2450G > A from a 4-year-old boy, and c.2090G > A and c.2122G > A from a 5-year-old boy), of which c.1_390del and c.2090G > A were novel. Functional studies revealed that the patient-derived lymphocytes carrying c.1_390del and c.2450G > A variants exhibited impaired mitochondrial function due to severe mitochondrial complexes I and III deficiencies, which was also found in *IARS2*-knockdown HEK293T cells. The compensatory experiments in *vitro* cell models confirmed the pathogenicity of *IARS2* variants since re-expression of wild-type *IARS2* rather than mutant *IARS2* could rescue complexes I and III deficiency, oxygen consumption, and cellular ATP content in *IARS2* knockdown cells.

**Conclusion:**

Our results not only expand the gene mutation spectrum of LS, but also reveal for the first time the pathogenic mechanism of *IARS2* variants due to a combined deficiency of mitochondrial complexes I and III, which is helpful for the clinical diagnosis of *IARS2* mutation-related diseases.

**Supplementary Information:**

The online version contains supplementary material available at 10.1186/s13023-024-03310-x.

## Introduction

Leigh syndrome (LS), also referred to as subacute necrotizing encephalopathy, is the most prevalent mitochondrial disease in childhood and is caused by mutations in both mitochondrial and nuclear genes [1]. LS was first reported by Denis Archibald Leigh in 1951 [2], with an estimated prevalence of 1:40,000 [3]. Although the incidence rate of LS is low, the clinical symptoms deteriorated rapidly and likely leading to death in infancy [4, 5]. LS is defined by the degeneration of the central nervous system, including the brain, spinal cord, and optic nerve, which is manifested by the loss of motor skills, appetite, vomiting, irritability, and/or seizures, as well as overall weakness, reduced muscle tone, and lactic acidosis, resulting in cardiac, hepatic, gastrointestinal and renal tubular dysfunction [4]. Currently, more than 100 pathogenic genes have been reported to be associated with LS [6]. LS is strongly associated with the oxidative phosphorylation system (OXPHOS) [7], and 80% of LS patients exhibit deficiencies of OXPHOS complexes [8]. While defects in each of the five OXPHOS complexes have been detected in LS patients, complex I deficiency is the most prevalent, with nearly a third of the LS causative genes linked to complex I deficiency [9, 10]. Isolated complex IV deficiency and multiple OXPHOS defects are commonly as well [11], providing evidence of biochemical defects to support a clinical diagnosis of LS.

*IARS2* encodes a mitochondrial isoleucyl-tRNA synthetase, which is a class I mitochondrial aminoacyl-tRNA synthetase (ARS) [12]. There are 37 members of the ARS family, which are associated with cytoplasmic or mitochondrial translation [13]. Mutations in genes encoding ARSs can produce highly diverse clinical phenotypes that affect tissues with particularly high metabolic needs [14]. As a nuclear-encoded mitochondrial protein, IARS2 needs to be imported from the cytoplasm into the mitochondria, and catalyzes the attachment of an isoleucine residue to a cognate mt-tRNA^Ile^ [15]. The patients with *IARS2* variants present broad clinical phenotypes including LS, West syndrome and CAGSSS syndrome (cataract, growth hormone deficiency, sensory neuropathy, sensorineural deafness-skeletal dysplasia syndrome) [14–18]. To date, 28 cases with *IARS2* mutations have been reported worldwide and 25 variants have been discovered [19], 14 of them have shown LS characteristic features [16, 17, 19–23]. However, the pathogenesis and molecular mechanism of the *IARS2* variants still unclear.

In this study, we reported two unrelated patients with LS harboring compound heterozygous *IARS2* variants (c.1_390del and c.2450G > A from a 4-year-old boy, and c.2090G > A and c.2122G > A from a 5-year-old boy) by next-generation sequencing. We applied patient-derived immortalized lymphocytes and *IARS2*-knockdown HEK293T cells to explore the pathogenesis of the variants.

## Materials and methods

### Patient recruitment and ethical considerations

The research and all associated procedures were granted approval by Peking University First Hospital (No. 2017–217). Before participating in the study, the patients and their parents provided informed consent. Subsequently, the patients were admitted to the Department of Pediatrics at Peking University First Hospital.

### Next-generation sequencing and variants analysis

The next-generation sequencing, including whole exome sequencing (WES), whole genome sequencing (WGS) and mitochondrial genomic sequencing, was performed using the Illumina HiSeq 2000 sequencer (Illumina, USA) [24]. The DNA was extracted from the peripheral blood of the patients and their parents. The sequence data was matched to the human reference genome (GRCh38/hg38). Germline short variants including SNPs, deletions or insertions were assessed using the genome analysis toolkit (GATK) [25]. WGS involved high-throughput sequencing of the entire genome and complete variant annotation. WES refered to the capture and enrichment of DNA from the exon regions of the entire genome for high-throughput sequencing. Pathogenic variants can be screened using the following criteria: (a) the inheritance pattern of the variants conforms to Mendel’s law of heredity; (b) in the population database, the allele frequency should be less than 1%; (c) pathogenicity analysis predicts that the mutations should be pathogenic or likely pathogenic; (d) there is a correlation between genotype and clinical phenotype. The identified variants were validated through Sanger sequencing.

To verify the variant site of patients, we extracted DNA using a small amount of genomic DNA extraction kit (Beyotime, China) form 1mL peripheral blood of the patients and their parents. The DNA was amplified using Taq Plus Polymerase (Vazyme, China) and then confirmed via Sanger sequencing. The amplification primers used were: Forward primer: 5′-CTGGCACTGCTGTGTGTCTA-3′; Reverse primer: 5′-AGTTCCAAACAAAATGTGTCTCA-3′.

### Pathogenicity analysis of variants

To verify the pathogenicity of *IARS2* variants at c.1_390del, c.2090G > A, c.2122G > A and c.2450G > A (GenBank Reference: NM_018060.4), we used ClustalW program to predict the conservation of sequences among different species [25]. We further investigated the potential pathogenicity of these variants by utilizing multiple population variation frequency and pathogenicity prediction databases, including gnomAD (https://gnomad.broadinstitute.org/) [26], dbSNP (https://www.ncbi.nlm.nih.gov/snp/) [27], ExAc (http://exac.broadinstitute.org) [28], Clinvar (https://www.ncbi.nlm.nih.gov/clinvar/) [29], Mutation Taster (https://www.mutationtaster.org/) [30] and PolyPhen2 (http://genetics.bwh.harvard.edu/pph2/) [31].

### Cell culture conditions

The peripheral blood cells from both patients and their parents were infected with Epstein–Barr virus generated from B95-8 cells (Cell Bank of the Chinese Academy of Sciences, China) to establish immortalized lymphocytes [32]. The immortalized lymphocytes were grown in Roswell Park Memorial Institute (RPMI) 1640 medium (Sigma-Aldrich, USA) supplemented with 10% fetal bovine serum (Sigma-Aldrich), 0.25 µg/mL amphotericin B (Beyotime Biotechnology), and 1% (v/v) penicillin-streptomycin (Beyotime Biotechnology). HEK293T cells (Cell Bank of the Chinese Academy of Sciences, Shanghai, China) were grown in Dulbecco’s Modified Eagle Medium (DMEM) medium (Sigma-Aldrich) supplemented with 12% cosmic calf serum (Sigma-Aldrich), 0.25 µg/mL amphotericin B, and 1% (v/v) penicillin-streptomycin. The cells were kept in an incubator (Thermo Fisher Scientific) with a 5% CO_2_ atmosphere at a temperature of 37 °C.

### Plasmid construct and site‑directed mutation

The short hairpin RNAs (shRNA) against *IARS2* were synthesized and cloned into the pLKO.1-puro vector (Vectorbuilder, Guangzhou, China). The coding sequences of *IARS2* (NM_018060) was synthesized and cloned into lentiviral vector, pLV [Exp]-EGFP: T2A: Puro‐EF1A (Cyagen, Guangdong, Guangzhou, China). Based on ClonExpress fast cloning technology, Site-specific mutation kit (Mut Express II Fast Mutagenesis Kit V2, Vacyme) was used to build site‑directed mutation according to the instructions. All primers were listed in Supplementary Table 1.

### Western blotting and blue native PAGE

For western blot, after collected and washed, cells were lysed with RIPA buffer (Cell Signaling Technology, USA) supplemented 1 mM PMSF (Sigma-Aldrich), then incubated the supernatant for 15 min and centrifuged at 12,000 × g for 10 min at 4 °C. The sample concentration was assessed using the Pierce BCA protein assay kit (Thermo Fisher Scientific). Subsequently, the protein sample was denatured with 5× loading buffer for 5 min at 95 °C. The separated samples were transferred onto a 0.22 μm polyvinylidene difluoride membrane (BIO-RAD, USA) after being separated using 10% bis-tris protein gels. The membrane was then blocked with 5% w/v milk and incubated with specific antibodies at room temperature for 1–2 h. Subsequently, the protein signals were visualized using Super-Signal West Pico Chemiluminescent Substrate (BIO-RAD). The primary antibodies used were anti-IARS2 (Proteintech, USA, 1:2000), anti-β-actin (Abcam, UK, 1:2000), anti-TOM70 (Abcam, UK, 1:2000), anti-rabbit IgG, HRP (Cell Signaling Technology, 1:2000), and anti-mouse IgG, HRP (Cell Signaling Technology, 1:2000).

For Blue native polyacrylamide gel electrophoresis (BN-PAGE) [33], cells were lysed using 20% Triton-100 (Sigma-Aldrich) for 20 min and centrifuged at 20,000 × g for 20 min at 4 °C. The samples were separated using 3.5-16% gradient polyacrylamide gels. The subsequent processes were the same as those for western blot. Five OXPHOS complexes were detected by anti-GRIM19 (Abcam, UK, 1:1000), anti-SDHA (Abcam, UK, 1:3000), anti-UQCRC2 (Abcam. UK, 1:2000), anti-MT-COI (Abcam, UK, 1:2000), and anti-ATP5A (Abcam, UK, 1:3000), respectively.

### Oxygen consumption rate

We employed the Oxygraph-2k system (Oroboros, Innsbruck, Austria) to assess the oxygen consumption of the cells [29]. Almost 1 × 10^7^ cells were seeded in cell-culture dish, after the cell density reaches about 80%, collected cells and washed them with PBS. Cells were gently and rapidly added to the machine. Following the measurement of basal respiration, 2 µg/mL oligomycin (Sigma-Aldrich) was administered to assess the ATP-linked mitochondrial respiration of the cells.

### Cellular adenosine triphosphate (ATP) detection

The cellular ATP levels were determined using an ATP bioluminescent assay kit (Sigma-Aldrich). Approximately 5 × 10^6^ cells were harvested, washed with PBS, and subsequently resuspended in an ATP-extracting solution (100 mM Tris-base, 4 mM EDTA-Na_2_, pH 7.75). Subsequently, the cells were boiled for 90 s, followed by centrifugation at 10,000 × g for 60 s to collect the supernatant. Finally, the supernatant was mixed with Luciferase Assay buffer, and the autofluorescence was detected. The results were corrected with protein concentration.

### Mitochondrial reactive oxygen species (ROS) detection

Mitochondrial ROS content was measured by using MitoSOX™ Red reagent (Thermo Fisher Scientific). Approximately 3 × 10^6^ cells were seeded in six-well plates. The cells were harvested and washed with PBS upon reaching approximately 80% cell density. Subsequently, the medium was replaced with a working solution containing 5 µM MitoSOX reagent. The cells were incubated at 37 °C for 10 min in the dark, gently collected, and washed with PBS. The fluorescence was determined using an excitation wavelength of 510 nm and an emission wavelength of 580 nm.

### Mitochondrial membrane potential (MMP) detection

MMP was measured by using tetramethylrhodamine (Thermo Fisher Scientific). For HEK293T cells, the six-well plates were seeded with nearly 3 × 10^6^ cells. The cells were washed with PBS upon reaching approximately 80% cell density. Then the medium was substituted with DMEM containing 30 nM tetramethylrhodamine. Subsequently, the cells were incubated at 37 °C for 30 min in the dark, gently collected, and washed with PBS three times. The fluorescence was then determined with excitation at 488 nm and emission at 570 nm. As for the immortalized lymphocytes, nearly 3 × 10^6^ cells were gathered, and the subsequent processes were the same as those for HEK293T cells.

### Statistical analysis

The quantitative experiments were conducted independently three times. The data were presented as the mean ± standard error of the mean (SEM). Statistical analyses were done using GraphPad Prism 8.0 (USA), and the independent Student’s t-test was utilized to calculate the *P*-values. A *P*-value of less than 0.05 was considered statistically significant (N.S. for *P* > 0.05, * for *P* < 0.05, ** for *P* < 0.01, *** for *P* < 0.001).

## Results

### Patient’s clinical features and genetics analysis

**Fig. 1:**
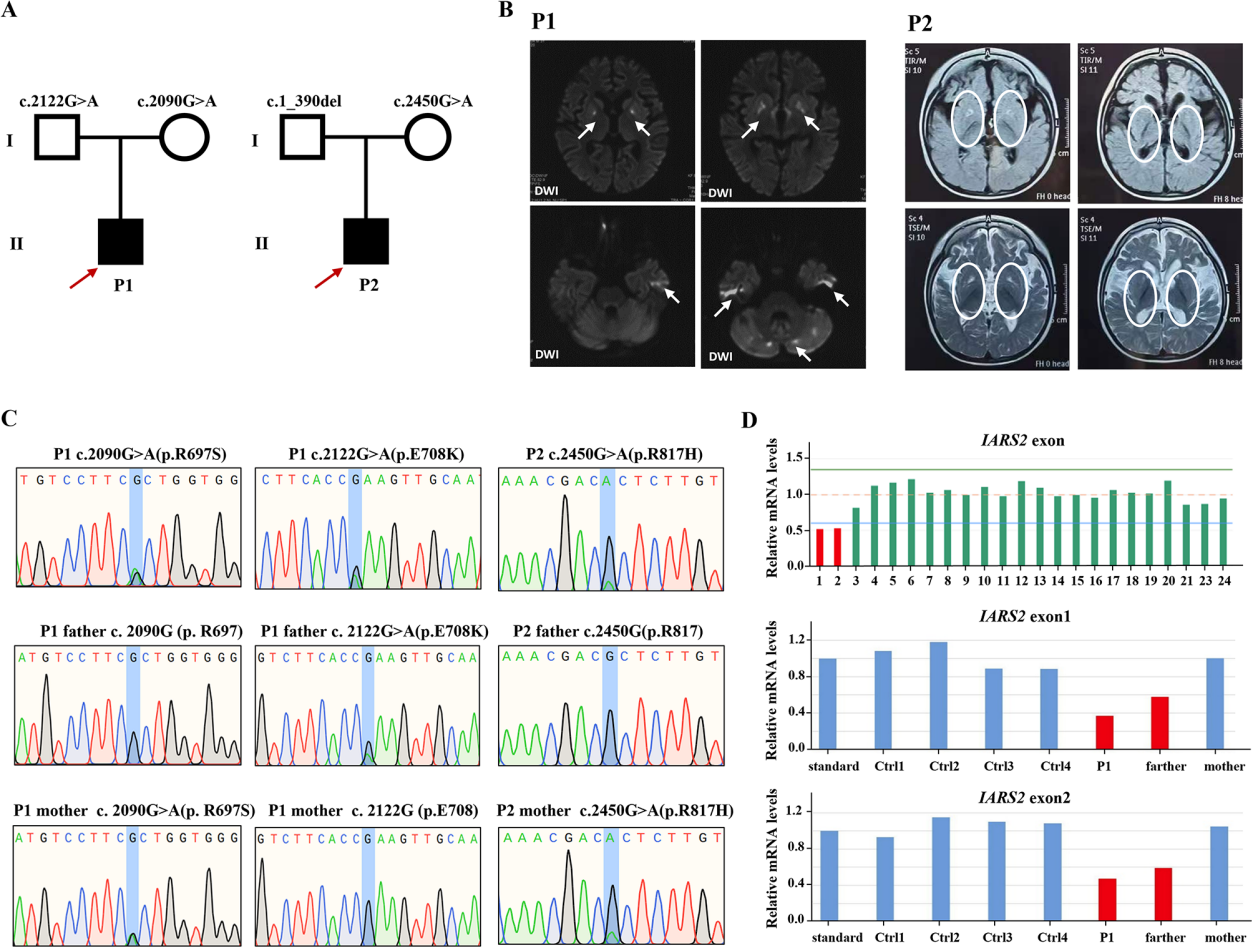
Family pedigree analysis, brain MRI images and variant analysis of *IARS2*. **A**. Pedigree analysis of patient 1 and patient 2 from two unrelated Chinese families. Rectangles indicated males, circles female, solid circle rectangled the affected individuals and probands were pointed out by arrows. **B**. Brain MRI images of patient 1 and 2. The patient 1 on the left showed lesions in the thalamus, periaqueductal, cerebellum and pons when he was 4 years old. The arrow pointed to the high signal were diseased areas. The patient 2 on the right showed long T1 and T2 signals in the lateral caudate nucleus and putamen, reduced cerebral white matter, bilateral basal ganglia symmetry damage, and cerebral dysplasia. The circled pointed to the symmetrical patchy were diseased areas. **C**. Sequencing chromatograms of *IARS2* in patients and their parents. The arrows indicated the mutation sites. **D**. Quantitative analysis results of *IARS2* in patient 2 and his parents

Patient 1, a 5-year-old boy, was born in a Chinese family with no family history of hereditary metabolic disease, with weighing 20 Kg, head circumference 52 cm and height 113 cm (Fig. 1A). He was G1P1 with a full-term normal delivery, and weighed 4000 g at birth. The patient frequently caught colds before the age of one and was admitted to the hospital at one and a half years old. Between the ages of 2 and 3 years, the patient’s feet were prone to peeling, and the symptom was relieved after vitamin B treatment. At 3 years and 10 months, the patient was readmitted with a high fever, weakness, and an inability to stand. The brain MRI diagnosed encephalitis. At 4 years, the patient’s brain MRI showed bilateral caudate nucleus and putamen nucleus were symmetrically swollen, and high diffusion weighted imaging (DWI) signal. (Fig. 1B) At 4 and a half years old, the patient experienced intermittent convulsions of both upper limbs, head, and face, and electroencephalography showed abnormal brain-wave activity. The patient’s brain MRI showed long T1 and T2 signals in the bilateral caudate nucleus and putamen nucleus, bilateral basal ganglia atrophy and brain sulci enlargement. Laboratory examination showed normal blood ammonia level (32 µM, normal range 18–60 µM). Whole exome sequencing (WES) and mitochondrial genomic sequencing were carried out to identify the disease-causing gene mutation. Following filtering with established criteria, two site variants (c.2090G > A and c.2122G > A) in the *IARS2* gene were found, and no clinically significant mitochondrial genomic-related variants were observed. Segregation analysis confirmed that c.2090G > A from the patient’s mother and c.2122G > A from the patient’s father (Fig. 1C).

Patient 2, a 4-year-old boy, was born in a Chinese family with healthy parents (Fig. 1A). He was G3P3 with an abdominal delivery, and weighed 4350 g at birth. The child was admitted to the hospital at one and a half years old, weighing 13 kg, with a head circumference of 46 cm and a height of 73 cm. He couldn’t speak and had been diagnosed with growth retardation. It was hard for him to sit stably, and he was unable to walk or eat alone. The patient was readmitted at age 3 and diagnosed with dystonia and neurodevelopmental delays. Laboratory examination showed increased blood lactate (6.24 mM, normal range 0.50–2.20 mM), β-hydroxybutyric acid (1.05 mM, normal range 0.02–0.27 mM), and creatine kinase (1854.59 U/L, normal range 50–319 U/L). Brain magnetic resonance imaging (MRI) revealed long T1 and T2 signals in the lateral caudate nucleus and putamen, reduced cerebral white matter, bilateral basal ganglia symmetry damage, and cerebral dysplasia (Fig. 1B). He was subsequently diagnosed with LS. After filtering with established criteria, WES and mitochondrial genomic sequencing did not detect potential disease-causing gene mutations. Subsequently, whole genome sequencing (WGS) was performed. Following filtering with established criteria, a novel site variant (c.2450G > A) and a deletion variant with 1 and 2 exons (c.1_390del, NC_000001.11: g.220267444_220269568del) in the *IARS2* gene were found, and no clinically significant mitochondrial genomic-related variants were observed. Segregation analysis confirmed that c.2450G > A was from the patient’s mother and c.1_390del was from the patient’s father (Fig. 1C). As shown in Fig. 1D, heterozygous mutations in *IARS2* exon 1 and 2 of the patient were found by CNV-Seq. Quantitative PCR results showed that the mRNA level of *IARS2* exon 1 and 2 in the patient and his father were significantly decreased compared with both age-matched controls and the patient’s mother.

### Pathogenicity analysis

**Fig. 2:**
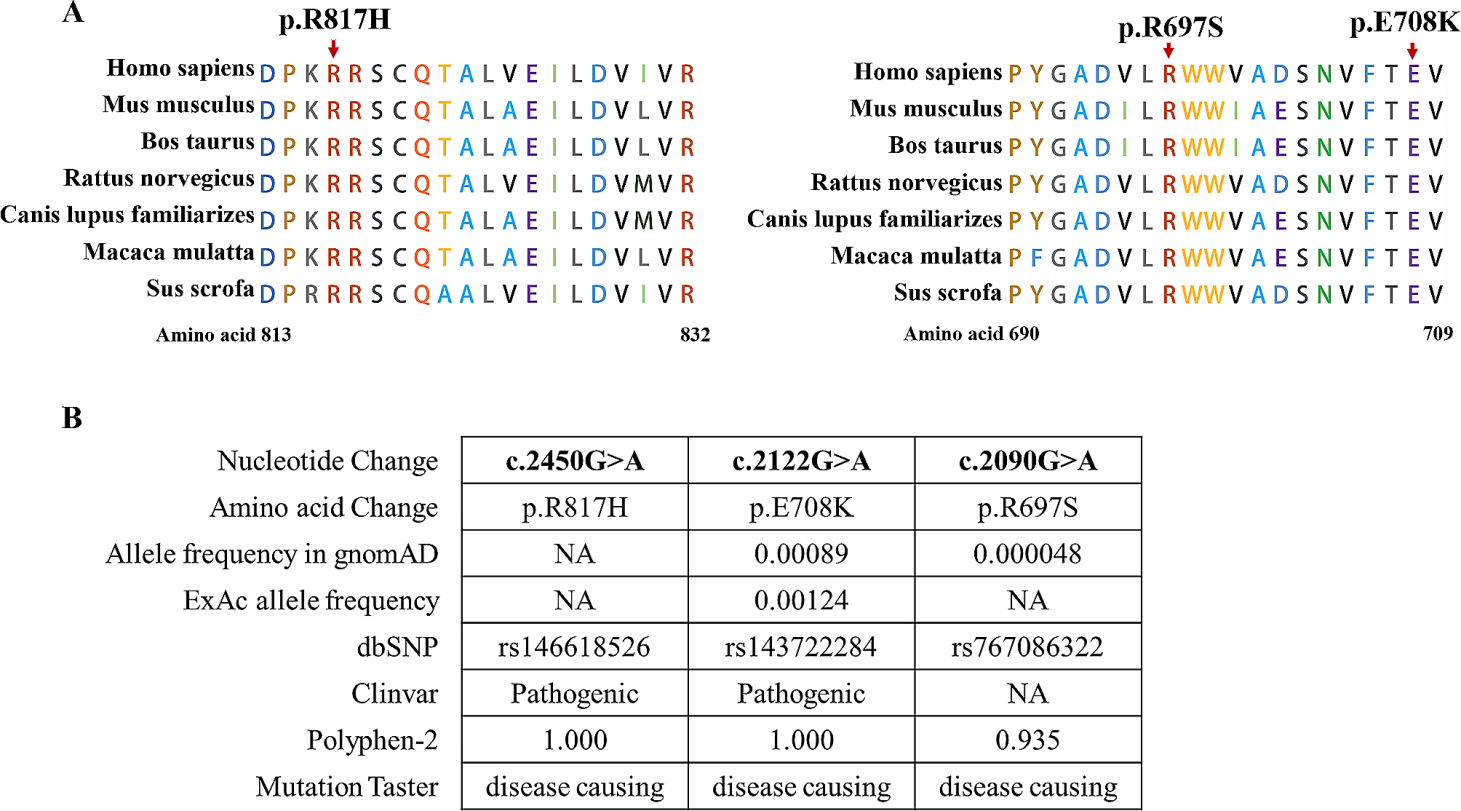
Conservative and pathogenicity analysis of *IARS2* variants. **A**. The conservation of amino acid of the mutation sites. **B**. Bioinformatic analysis of allele frequency and pathogenicity prediction about three variants (c.2450G > A, c.2122G > A and c.2090G > A)

We further investigated the pathogenicity of the above *IARS2* variants (c.2450G > A, c.2122G > A and c.2090G > A). Firstly, conservation analysis of amino acid indicated that c.2450G > A (p.R817H), c.2122G > A (p.E708K) and c.2090G > A (p.R697S) were highly conservation among difference species (Fig. 2A). Furthermore, we performed allele frequency analysis of these variants. The allele frequency of c.2122G > A and c.2090G > A in gnomAD was extremely low, with 0.00089 and 0.00048, respectively. The other variants have not been recorded. According to Mutation Taster, the c.2450G > A (p.R817H), c.2122G > A (p.E708K), and c.2090G > A (p.R697S) variants were predicted to be disease-causing. The prediction results of PolyPhen2 also indicated that these variants were pathogenic mutations, with a numerical score of 1.0 for c.2450G > A (p.R817H), 1.0 for c.2122G > A (p.E708K), and 0.935 for c.2090G > A (p.R697S) (Fig. 2B). A score range of 0.0 to 1.0 indicated the likelihood of benign to harmful effects. These results suggested that the c.2450G > A, c.2122G > A and c.2090G > A variants were likely to be disease-causing.

### Mitochondrial function was impaired in patient-derived lymphocytes

**Fig. 3:**
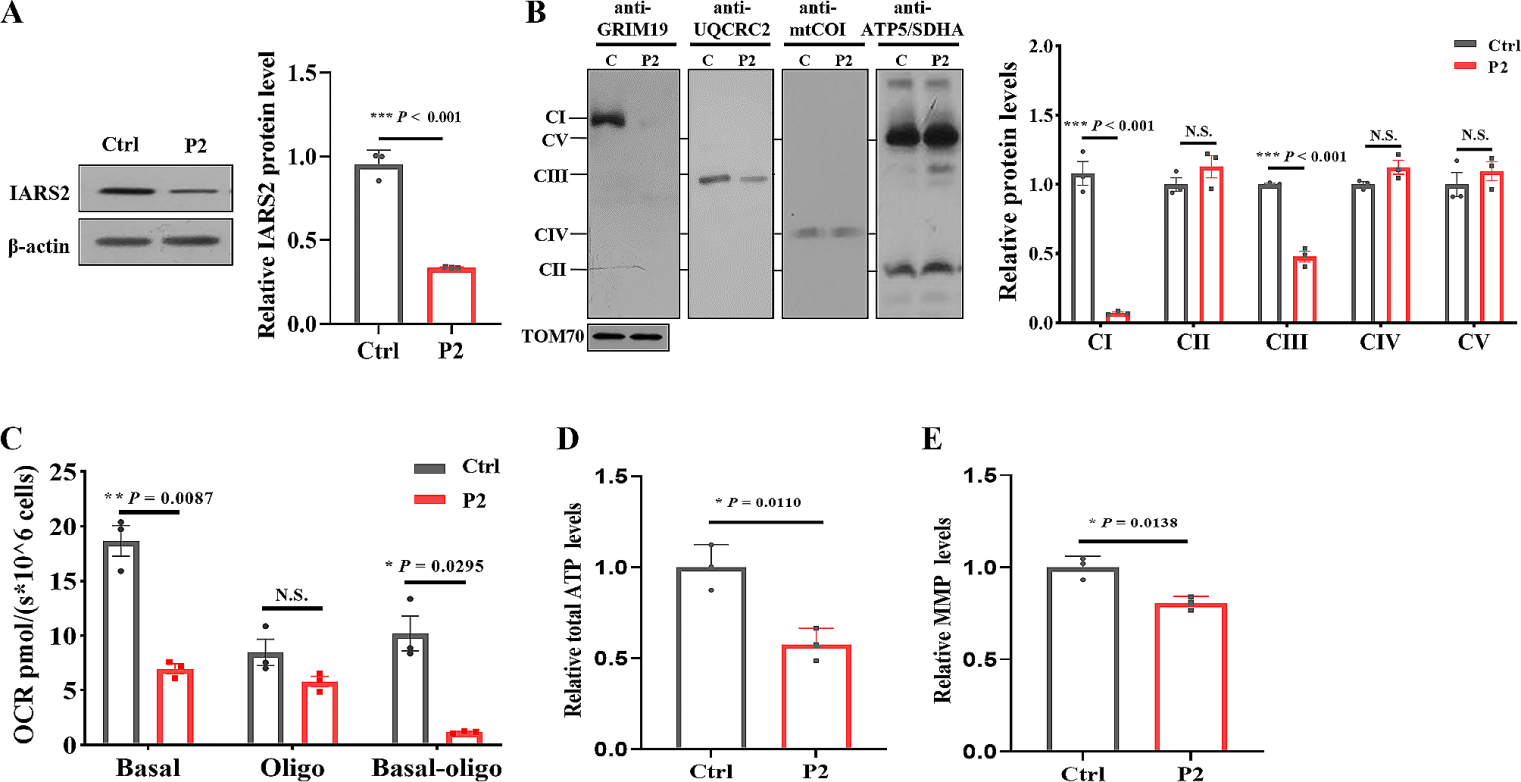
IARS2 expression and mitochondrial functional validation in patient-derived lymphocytes with c.1_390del and c.2450G > A variants. **A**. Immunoblot and quantification analysis of IARS2 protein in healthy age-matched control and patient-derived lymphocytes. β‐actin was used as control. **B**. BN-PAGE/immunoblot and quantification analysis for OXPHOS complexes in healthy age-matched control and patient-derived lymphocytes. TOM70 was used as control. **C**. Oxygen consumption rate in healthy age-matched control and patient-derived lymphocytes by using Oxygraph‐2k. Basal, basal respiration; Oligo, ATP-linked mitochondrial respiration of the cells added with 2 µg/mL oligomycin. **D-E**. Relative cellular ATP (**D**) and MMP (**E**) content in healthy age-matched control and patient-derived lymphocytes. N.S. *P* > 0.05, * *P* < 0.05, *** *P* < 0.001

To determine the pathogenicity of *IARS2* variants, we constructed immortalized lymphocytes derived from patient 2. However, due to physical condition of patient 1, we were unable to establish immortalized lymphocytes from this patient. As shown in Fig. 3A, the IARS2 protein level of patient-derived immortalized lymphocytes was significantly decreased compared with age-matched control cells. We further investigated the OXPHOS complexes in patient‐derived immortalized lymphocytes, the results indicated that the contents of complexes I and III were decreased by ~ 90% and ~ 50% compared with control cells, respectively (Fig. 3B). Patient-derived immortalized lymphocytes showed a significant reduction in basal respiration and ATP-linked respiration compared to control cells (Fig. 3C). In order to further explore the effect of *IARS2* variants on mitochondrial function, we measured the cellular ATP level and MMP content, and results showed that the production of cellular ATP and MMP was decreased compared with control cells (Fig. 3D and E). Altogether, these results indicated that patient‐derived immortalized lymphocytes had impaired mitochondrial function.

### *IARS2* deficiency caused mitochondrial dysfunction

**Fig. 4:**
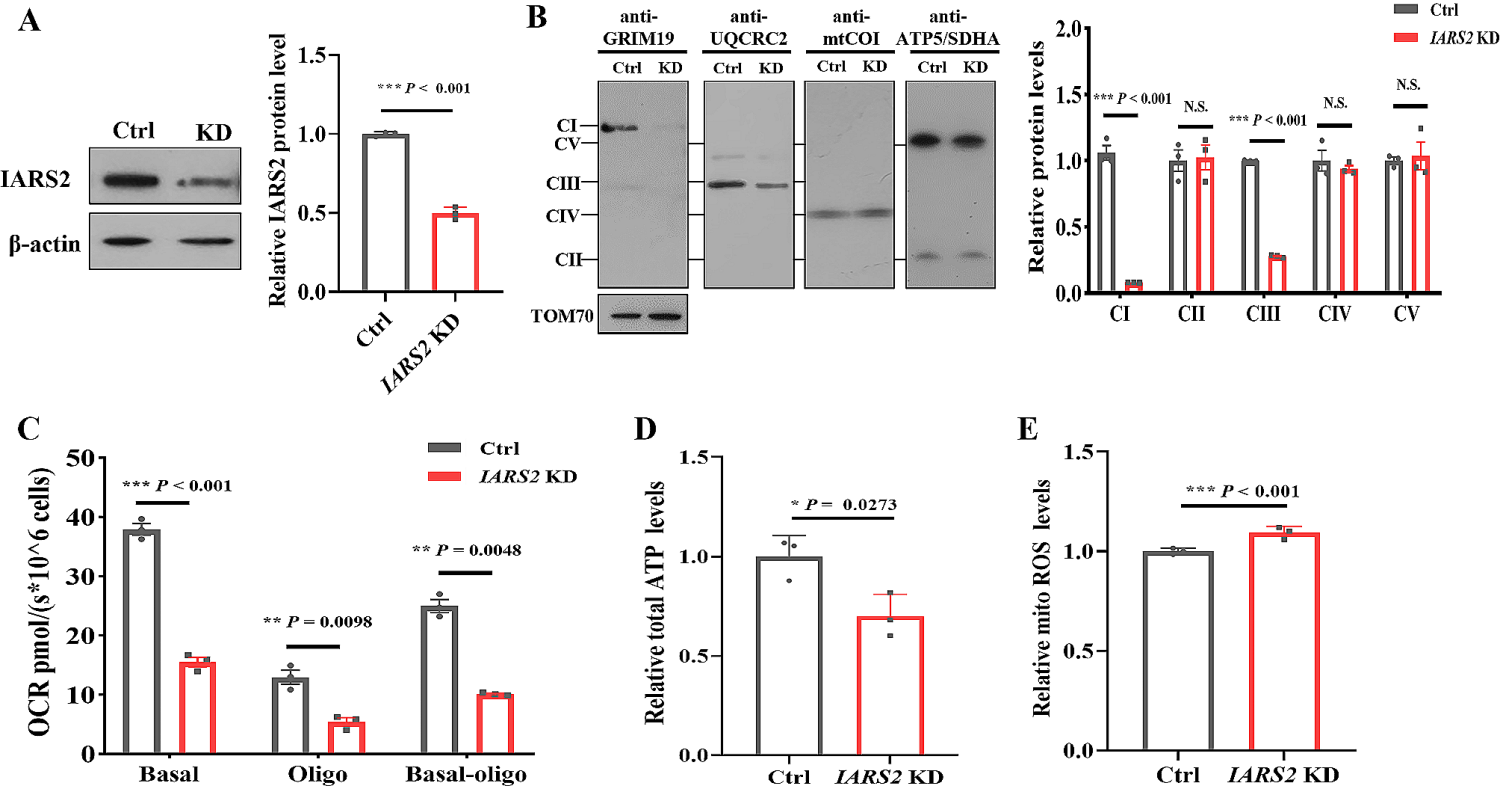
Mitochondrial function was impaired in *IARS2* knockdown cells. **A**. Immunoblot and quantification analysis of IARS2 protein in control and *IARS2* knockdown cells. β-actin was used as control. **B**. BN-PAGE/immunoblot analysis and quantification for OXPHOS complexes in control and *IARS2* knockdown cells. TOM70 was used as control. **C**. Oxygen respiration rate in control and *IARS2* knockdown cells by using Oxygraph-2k. Basal, basal respiration; Oligo, ATP-linked mitochondrial respiration of the cells added with 2 µg/mL oligomycin. **D-E**. Relative cellular ATP content (**D**) and mitochondrial ROS level (**E**) of control and *IARS2* knockdown cells. N.S. *P* > 0.05, * *P* < 0.05, ** *P* < 0.01, *** *P* < 0.001

To verify the pathogenicity of *IARS2* deficiency, shRNA-mediated *IARS2* knockdown was established in HEK293T cells, with an 60% decrease in the expression of IARS2 protein (Fig. 4A). In agreement with patient-derived immortalized lymphocytes, the contents of OXPHOS complexes I and III were markedly decreased in *IARS2* knockdown cells than that in control cells (Fig. 4B). Basal respiration and ATP-linked respiration were also significantly impaired in *IARS2* knockdown cells compared with control cells (Fig. 4C). Furthermore, the production of cellular ATP was notably decreased and the mitochondrial ROS level was increased in *IARS2* knockdown cells compared with control cells (Fig. 4D, E). These results collectively indicated that *IARS2* was indispensable for the maintenance of mitochondrial function, and *IARS2* deficiency caused mitochondrial dysfunction by impairing complexes I and III.

### *IARS2* variants at c.2090G > A, c.2122G > A and c.2450G > A led to mitochondrial dysfunction

**Fig. 5:**
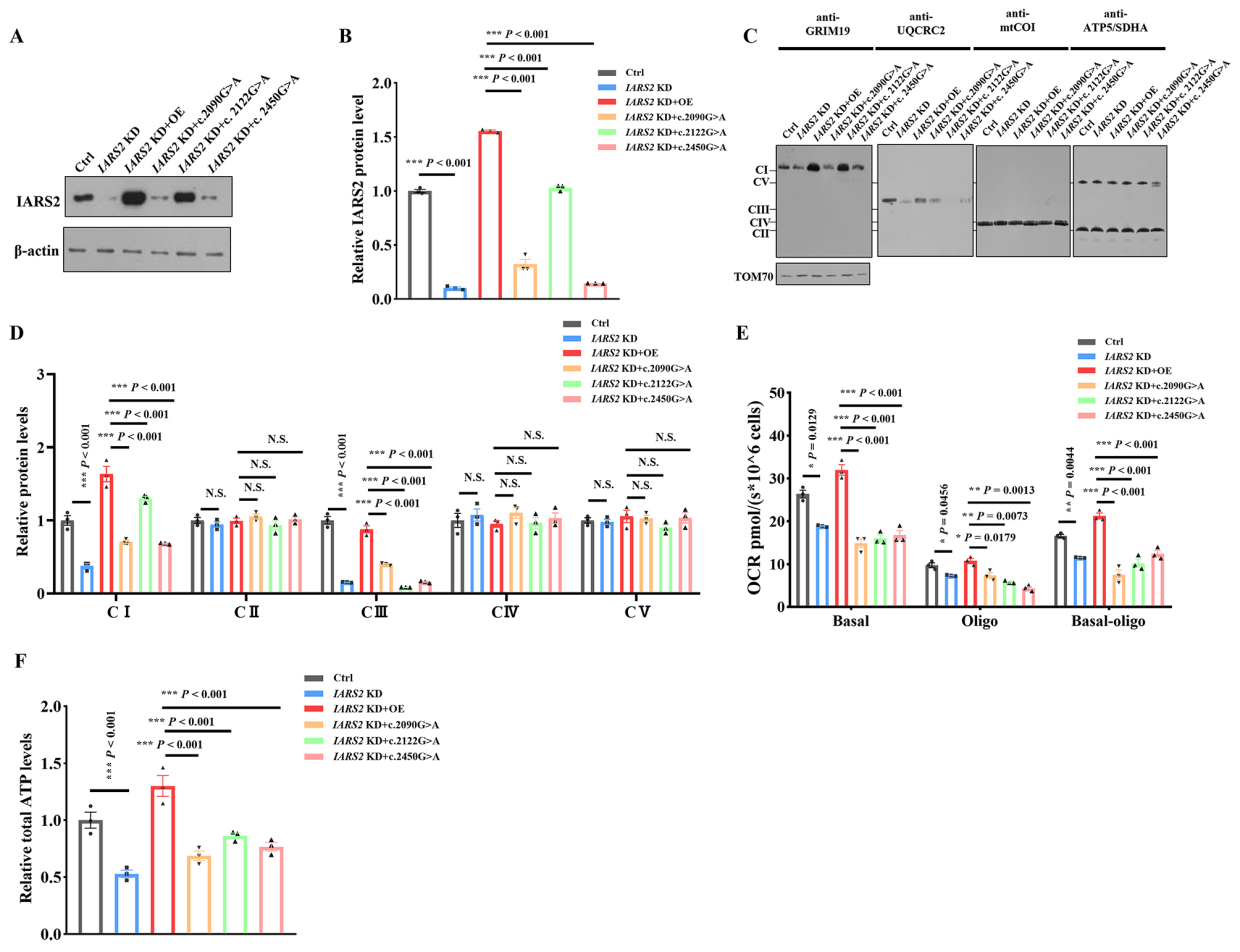
*IARS2* variants at c.2090G > A, c.2122G > A and c.2450G > A led to mitochondrial dysfunction. **A-B**. Immunoblot (**A**) and quantification (**B**) analysis of IARS2 protein in *IARS2* knockdown HEK293T cells with re-expression of wild-type IARS2 or mutant *IARS2*. β‐actin was used as control. **C-D**. BN-PAGE/immunoblot (**C**) and quantification (**D**) analysis for OXPHOS complexes in *IARS2* knockdown HEK293T cells with re-expression of re-expressed wild-type *IARS2* or mutant *IARS2*. TOM70 was used as control. **E**. Oxygen respiration rate of *IARS2* knockdown HEK293T cells with re-expression of wild-type *IARS2* or mutant *IARS2*. Basal, basal respiration; Oligo, ATP-linked mitochondrial respiration of the cells added with 2 µg/mL oligomycin. **F**. Relative cellular ATP content of with re-expression of wild-type *IARS2* or mutant *IARS2* carrying c.2090G > A, c.2122G > A or c.2450G > A in *IARS2* knockdown HEK293T cells. N.S. *P* > 0.05, * *P* < 0.05, ** *P* < 0.01, *** *P* < 0.001

To further verify the pathogenicity of these *IARS2* variants, we re-expressed wild-type *IARS2* and mutant *IARS2* carrying c.2090G > A, c.2122G > A or c.2450G > A variants in cells with *IARS2* knockdown. Re‐expression of wild‐type *IARS2* in *IARS2* knockdown cells significantly increased the level of IARS2 protein. However, the levels of IARS2 protein in re-expressed mutant *IARS2* cells carrying c.2090G > A, c.2122G > A and c.2450G > A were all significantly lower than that in wild-type *IARS2* cells (Fig. 5A), suggesting that c.2090G > A, c.2122G > A and c.2450G > A variants could cause IARS2 deficiency. We further tested the OXPHOS complexes and mitochondrial function. As shown in Fig. 5B and C, OXPHOS complexes I and III, basal respiration and ATP-linked respiration were impaired compared with cells of re-expressed wild‐type *IARS2*. The production of cellular ATP was also decreased in HEK293T cells re‐expressing of mutant *IARS2* carrying c.2090G > A, c.2122G > A and c.2450G > A relative to HEK293T cells with re‐expressing wild‐type *IARS2* (Fig. 5D). Altogether, these results suggested that c.2090G > A, c.2122G > A and c.2450G > A variants in *IARS2* were pathogenic and led to mitochondrial dysfunction.

## Discussion

In this study, we identified four heterozygous variants of *IARS2* (c.1_390del and c.2450G > A, c.2122G > A and c.2090G > A) from two unrelated Chinese patients of LS, of which c.1_390del and c.2090G > A were novel. Functional studies showed these *IARS2* variants could result in IARS2 deficiency, which in turn led to mitochondrial dysfunction due to a combined deficiency of complexes I and III. Our study revealed that *IARS2* was essential for mitochondrial OXPHOS function, particularly in the maintenance of OXPHOS complexes I and III. To the best of our knowledge, this is the first report to elucidate the pathogenesis of *IARS2* variants.

Although exons account for less than 2% of the human genome, they contain over 85% of known pathogenic mutations. WES is currently the most widely used DNA sequencing technology in clinical practice due to its cost-effectiveness and high sequencing depth. However, WES focuses solely on the protein-coding regions, including exons and adjacent intron/exon boundaries, and is unable to detect large fragment indels. In contrast, WGS analyzes the entire genome, encompassing nearly all exon sequences of genes, as well as intron sequences, intergenic regions, and can simultaneously detect mtDNA variations. Moreover, it can identify genomic structural variations such as large fragment deletions, copy number variations, and other gene structural alterations. If WES and mitochondrial genome sequencing fail to detect potential pathogenic gene mutations, WGS can be utilized for reassessment to uncover possible pathogenic gene mutations. In this study, we reported two unrelated children with LS. For patient 1, compound heterozygous *IARS2* variants (c.2090G > A and c.2122G > A) were identified by WES, and no clinically significant mitochondrial genomic-related variants were observed. For patient 2, after filtering with established criteria, WES and mitochondrial genomic sequencing did not detect potential disease-causing gene mutations. Subsequently, whole genome sequencing (WGS) was performed, revealing compound heterozygous *IARS2* variants, a deletion variant spanning 1 and 2 exons (c.1_390del), and a site variant c.2450G > A. This demonstrates that WGS has further improved the efficiency of disease diagnosis.

Three patients with *IARS2* variants of CAGSSS syndrome were first reported in 2014 [16]. To date, 28 cases with *IARS2* variants from 10 different countries have been reported. Among these cases, 13 patients exhibited short stature, 4 had hypoparathyroidism, 19 had elevated lactate levels in the blood or cerebrospinal fluid, 6 patients had sideroblastic anemia, 5 patients had heart problems, and 14 patients had abnormalities on their brain MRI [16, 17, 19–23, 34]. According to the clinical phenotype, 12 were diagnosed with LS, 7 were diagnosed with CAGSSS, 4 were diagnosed with West syndrome, 2 showed both LS and CAGSSS features, 6 were diagnosed with sideroblastic anemia, and 1 was diagnosed with Wolff-Parkinson-White syndrome [16, 17, 19–23, 34]. It seems that LS and CAGSSS are more common in patients with *IARS2* gene variants.

To date, there are 25 reported disease-linked variants have been described in *IARS2* gene. Of these, only the *IARS2* c.2726 C > T (GenBank Reference: NM_018060.3) and c.2725 C > T (GenBank Reference: NM_018060.3) variants in *IARS2* were functionally tested. IARS2 protein level were decreased in patient-derived fibroblasts with c.2726 C > T variant compared with control cells, but there was no significant difference in the contents of OXPHOS complexes between patient and control fibroblasts [16]. However, IARS2 protein level and OXPHOS complexes levels did not change in the patient‐derived fibroblasts with c.2725 C > T variant compared with control cells [34]. In this study, we reported two unrelated patients who presented with LS and had biallelic variants (c.1_390del and c.2450G > A, c.2122G > A and c.2090G > A) in *IARS2*. Among these variants, the variants c.2122G > A and c.2450G > A were previously reported in a patient diagnosed with LS and in a family with two Japanese siblings who exhibited milder symptoms of CAGSSS and West syndrome alongside LS, respectively. However, functional tests had not been conducted in previous studies [16, 17]. Here, our study revealed that the patient-derived lymphocytes carrying c.1_390del and c.2450G > A variants exhibited decreased IARS2 protein level and impaired mitochondrial function due to the deficiency of OXPHOS complexes I and III contents. We further knockdown *IARS2* and found that *IARS2* deficiency could cause decreased complexes I and III contents, impaired basal respiration and ATP-linked respiration and reduced cellular ATP production, suggesting that *IARS2* deficiency led to mitochondrial dysfunction. To verify the pathogenicity of these *IARS2* variants, we re-expressed wild‐type *IARS2* and mutant *IARS2* with c.2450G > A, c.2122G > A or c.2090G > A variants in cells with *IARS2* knockdown, respectively. The results showed that IARS2 protein levels were damaged with mutant *IARS2*, and contents of OXPHOS complex I and III, basal respiration, ATP-linked respiration and cellular ATP production were also impaired. These data suggested that these *IARS2* variants were pathogenic and could cause mitochondrial dysfunction due to the decreased contents of mitochondrial OXPHOS complexes I and III.

In summary, we report two unrelated Chinese patients who manifested with LS carrying biallelic *IARS2* variants (c.1_390del and c.2450G > A, c.2122G > A and c.2090G > A), and reveal that *IARS2* deficiency cause combined complexes I and III deficiencies and mitochondrial dysfunction. Our study further expands the gene mutation spectrum of LS, and uncovers that *IARS2* is indispensable for the maintenance of mitochondrial complexes I and III, which provides the pathogenic mechanism and functional evidence of *IARS2* as a gene causing mitochondrial diseases and contributes to the clinical diagnosis of *IARS2* mutation-related diseases.

### Electronic supplementary material

Below is the link to the electronic supplementary material.

Supplementary Material 1

## Data Availability

Data cannot be made publicly available due to ethical reasons. Researchers interested in getting access to the data should feel free to contact the corresponding author (yawang@wmu.edu.cn).

## References

[1] Stenton SL, Zou Y, Cheng H, Liu Z, Wang J, Shen D, Jin H, Ding C, Tang X, Sun S. Leigh syndrome: a study of 209 patients at the Beijing children’s hospital. Ann Neurol. 2022;91(4):466–82. 35094435 10.1002/ana.26313

[2] Leigh D. Subacute necrotizing encephalomyelopathy in an infant. J Neurol Neurosurg Psychiatry. 1951;14(3):216. 14874135 10.1136/jnnp.14.3.216 PMC499520

[3] Yahya V, Spagnolo F, Di Maggio G, Leopizzi E, De Marco P, Fortunato F, Comi GP, Rini A, Monfrini E, Di Fonzo A. Juvenile-onset dystonia with spasticity in Leigh syndrome caused by a novel NDUFA10 variant. Parkinsonism Relat Disord. 2022;104:85–7. 36270260 10.1016/j.parkreldis.2022.10.016

[4] Lim AZ, Ng YS, Blain A, Jiminez-Moreno C, Alston CL, Nesbitt V, Simmons L, Santra S, Wassmer E, Blakely EL. Natural history of Leigh syndrome: a study of disease burden and progression. Ann Neurol. 2022;91(1):117–30. 34716721 10.1002/ana.26260 PMC9534328

[5] Kistol D, Tsygankova P, Krylova T, Bychkov I, Itkis Y, Nikolaeva E, Mikhailova S, Sumina M, Pechatnikova N, Kurbatov S. Leigh syndrome: spectrum of molecular defects and clinical features in Russia. Int J Mol Sci. 2023;24(2):1597. 36675121 10.3390/ijms24021597 PMC9865855

[6] of Neurology SG, of, Neurology SC, Board E. Expert consensus on the diagnosis and treatment of Leigh syndrome (2023). Zhonghua er ke za zhi = Chinese J Pediatrics. 2023;61(12):1077–1085. 10.3760/cma.j.cn112140-20230904-00153 38018044

[7] Fernandez-Vizarra E, Zeviani M. Mitochondrial disorders of the OXPHOS system. FEBS Lett. 2021;595(8):1062–106. 33159691 10.1002/1873-3468.13995

[8] Chang X, Wu Y, Zhou J, Meng H, Zhang W, Guo J. A meta-analysis and systematic review of Leigh syndrome: clinical manifestations, respiratory chain enzyme complex deficiency, and gene mutations. Medicine. 2020;99(5). 10.1097/MD.0000000000018634 PMC7004636 32000367

[9] Rahman S. Leigh syndrome. Handbook of clinical neurology. Volume 194. Elsevier; 2023. pp. 43–63. 10.1016/B978-0-12-821751-1.00015-4 36813320

[10] Zhou X, Lou X, Zhou Y, Xie Y, Han X, Dong Q, Ying X, Laurentinah MR, Zhang L, Chen Z. Novel biallelic mutations in TMEM126B cause splicing defects and lead to Leigh-Like syndrome with severe complex I deficiency. J Hum Genet. 2023;68(4):239–46. 36482121 10.1038/s10038-022-01102-4 PMC10040336

[11] Lim SC, Smith KR, Stroud DA, Compton AG, Tucker EJ, Dasvarma A, Gandolfo LC, Marum JE, McKenzie M, Peters HL. A founder mutation in PET100 causes isolated complex IV deficiency in Lebanese individuals with Leigh syndrome. Am J Hum Genet. 2014;94(2):209–22. 24462369 10.1016/j.ajhg.2013.12.015 PMC3928654

[12] Bonnefond L, Fender A, Rudinger-Thirion J, Giegé R, Florentz C, Sissler M. Toward the full set of human mitochondrial aminoacyl-tRNA synthetases: characterization of AspRS and TyrRS. Biochemistry. 2005;44(12):4805–16. 15779907 10.1021/bi047527z

[13] Antonellis A, Green ED. The role of aminoacyl-tRNA synthetases in genetic diseases. Annu Rev Genomics Hum Genet. 2008;9:87–107. 18767960 10.1146/annurev.genom.9.081307.164204

[14] Meyer-Schuman R, Antonellis A. Emerging mechanisms of aminoacyl-tRNA synthetase mutations in recessive and dominant human disease. Hum Mol Genet. 2017;26(R2):R114–27. 28633377 10.1093/hmg/ddx231 PMC5886470

[15] Florentz C, Sohm B, Tryoen-Toth P, Pütz J, Sissler M. Human mitochondrial tRNAs in health and disease. Cell Mol Life Sci CMLS. 2003;60:1356–75. 12943225 10.1007/s00018-003-2343-1 PMC11138538

[16] Schwartzentruber J, Buhas D, Majewski J, Sasarman F, Papillon-Cavanagh S, Thiffaut I, Sheldon KM, Massicotte C, Patry L, Simon M. Mutation in the Nuclear‐Encoded mitochondrial Isoleucyl–t RNA synthetase IARS2 in patients with cataracts, growth hormone Deficiency with short stature, partial Sensorineural Deafness, and Peripheral Neuropathy or with Leigh Syndrome. Hum Mutat. 2014;35(11):1285–9. 25130867 10.1002/humu.22629

[17] Takezawa Y, Fujie H, Kikuchi A, Niihori T, Funayama R, Shirota M, Nakayama K, Aoki Y, Sasaki M, Kure S. Novel IARS2 mutations in Japanese siblings with CAGSSS, Leigh, and West syndrome. Brain Develop. 2018;40(10):934–8. 10.1016/j.braindev.2018.06.010 30041933

[18] Moosa S, Haagerup A, Gregersen PA, Petersen KK, Altmüller J, Thiele H, Nürnberg P, Cho TJ, Kim OH, Nishimura G. Confirmation of CAGSSS syndrome as a distinct entity in a Danish patient with a novel homozygous mutation in IARS2. Am J Med Genet Part A. 2017;173(4):1102–8. 28328135 10.1002/ajmg.a.38116

[19] Gong Y, Lan XP, Guo S. IARS2-related disease manifesting as sideroblastic anemia and hypoparathyroidism: a case report. Front Pead. 2023;10:1080664. 10.3389/fped.2022.1080664 PMC9871752 36704128

[20] Upadia J, Li Y, Walano N, Deputy S, Gajewski K, Andersson HC. Genotype–phenotype correlation in IARS2-related diseases: a case report and review of literature. Clin Case Rep. 2022;10(2):e05401. 35228874 10.1002/ccr3.5401 PMC8867157

[21] Barcia G, Pandithan D, Ruzzenente B, Assouline Z, Pennisi A, Ormieres C, Besmond C, Roux C-J, Boddaert N, Desguerre I. Biallelic IARS2 mutations presenting as sideroblastic anemia. Haematologica. 2021;106(4):1220. 33327715 10.3324/haematol.2020.270710 PMC8018106

[22] Roux C-J, Barcia G, Schiff M, Sissler M, Levy R, Dangouloff-Ros V, Desguerre I, Edvardson S, Elpeleg O, Rötig A. Phenotypic diversity of brain MRI patterns in mitochondrial aminoacyl-tRNA synthetase mutations. Mol Genet Metab. 2021;133(2):222–9. 33972171 10.1016/j.ymgme.2021.04.004

[23] Lee JS, Kim MJ, Kim SY, Lim BC, Kim KJ, Choi M, Seong M-W, Chae J-H. Clinical and genetic characteristics of Korean patients with IARS2-related disorders. J Genetic Med. 2019;16(2):55–61. 10.5734/JGM.2019.16.2.55

[24] Calvo SE, Compton AG, Hershman SG, Lim SC, Lieber DS, Tucker EJ, Laskowski A, Garone C, Liu S, Jaffe DB. Molecular diagnosis of infantile mitochondrial disease with targeted next-generation sequencing. Sci Transl Med. 2012;4(118):ra118110–118110. 10.1126/scitranslmed.3003310 PMC3523805 22277967

[25] Thompson JD, Gibson TJ, Higgins DG. Multiple sequence alignment using ClustalW and ClustalX. Curr Protocols Bioinf. 2003(1):2.3. 1–2.3. 22. 10.1002/0471250953.bi0203s00 18792934

[26] Gudmundsson S, Singer-Berk M, Watts NA, Phu W, Goodrich JK, Solomonson M, Consortium GAD, Rehm HL. MacArthur DG, O’Donnell‐Luria A: variant interpretation using population databases: lessons from gnomAD. Hum Mutat. 2022;43(8):1012–30. 34859531 10.1002/humu.24309 PMC9160216

[27] Sherry ST, Ward M-H, Kholodov M, Baker J, Phan L, Smigielski EM, Sirotkin K. dbSNP: the NCBI database of genetic variation. Nucleic Acids Res. 2001;29(1):308–11. 11125122 10.1093/nar/29.1.308 PMC29783

[28] Kobayashi Y, Yang S, Nykamp K, Garcia J, Lincoln SE, Topper SE. Pathogenic variant burden in the ExAC database: an empirical approach to evaluating population data for clinical variant interpretation. Genome Med. 2017;9(1):1–14. 28166811 10.1186/s13073-017-0403-7 PMC5295186

[29] Landrum MJ, Lee JM, Benson M, Brown GR, Chao C, Chitipiralla S, Gu B, Hart J, Hoffman D, Jang W. ClinVar: improving access to variant interpretations and supporting evidence. Nucleic Acids Res. 2018;46(D1):D1062–7. 29165669 10.1093/nar/gkx1153 PMC5753237

[30] Schwarz JM, Rödelsperger C, Schuelke M, Seelow D. MutationTaster evaluates disease-causing potential of sequence alterations. Nat Methods. 2010;7(8):575–6. 20676075 10.1038/nmeth0810-575

[31] Adzhubei I, Jordan DM, Sunyaev SR. Predicting functional effect of human missense mutations using PolyPhen-2. Curr Protocols Hum Genet. 2013;76(1):7. 21–27.20. 41. 10.1002/0471142905.hg0720s76 PMC4480630 23315928

[32] Zhou X, Lou X, Zhou Y, Xie Y, Han X, Dong Q, Ying X, Laurentinah MR, Zhang L, Chen Z. Novel biallelic mutations in TMEM126B cause splicing defects and lead to Leigh-Like syndrome with severe complex I deficiency. J Hum Genet. 2022:1–8. 10.1038/s10038-022-01102-4 PMC10040336 36482121

[33] Wittig I, Braun H-P, Schägger H. Blue native PAGE. Nat Protoc. 2006;1(1):418–28. 17406264 10.1038/nprot.2006.62

[34] Vona B, Maroofian R, Bellacchio E, Najafi M, Thompson K, Alahmad A, He L, Ahangari N, Rad A, Shahrokhzadeh S. Expanding the clinical phenotype of IARS2-related mitochondrial disease. BMC Med Genet. 2018;19:1–16. 30419932 10.1186/s12881-018-0709-3 PMC6233262

