# Supplementary material for: IARS2 mutations lead to Leigh syndrome with a combined oxidative phosphorylation deficiency
Source: Orphanet J Rare Dis. 2024 Aug 21;19:305. doi: 10.1186/s13023-024-03310-x (PMC11340112; doi:10.1186/s13023-024-03310-x)
Supplement: Supplementary file 1 — Supplementary Material 1 [file 13023_2024_3310_MOESM1_ESM.docx]

**Supplemental Table S1. Sequences of primers used in this study.**

| **Sequences of primers used for vector construction** | |
| --- | --- |
| *IARS2* overexpression | F:5'- atttccggtgaattcctcgagATGCGTTGGGGGCTGCGC -3' |
|  | R:5'-gagggagaggggcgggatccCTATTTTCgCACTGACAACTTCTG  CA -3' |
| **Sequences of primers used for SNP site**-**directed mutagenesis** | |
| *IARS2* c.2090G>A | F:5'- ATGTCCTTC**A**CTGGTGGGTAGCTGATTCCAAT -3' |
|  | R:5'- CCACCAG**T**GAAGGACATCAGCACCATACGGAG -3' |
| *IARS2* c.2122G>A | F:5'- GTCTTCACC**A**AAGTTGCAATTGGCCCATCCGT -3' |
|  | R:5'- GCAACTT**T**GGTGAAGACATTGGAATCAGCTACC -3' |
| *IARS2* c.2450G>A | F:5'- CAAACGAC**A**CTCTTGTCAGACTGCATTAGTTGAAA -3' |
|  | R:5'- GACAAGAG**T**GTCGTTTGGGGTCATTTTCCTTT -3' |
| **Sequences of shRNA** | |
| *IARS2* shRNA-1 | F:5'-ccggGAGCATGTCAGTCGTTCAATActcgagTATTGAACGA  CTGACATGCTCtttttg-3' |
|  | R:5'-aattcaaaaaGAGCATGTCAGTCGTTCAATActcgagTATTGAA  CGACTGACATGCTC-3' |
| *IARS2* shRNA-2 | F:5'-ccggCTACGTACTGGCGGCAGATAActcgagTTATCTGCCG  CCAGTACGTAGtttttg-3' |
|  | R:5'-aattcaaaaaCTACGTACTGGCGGCAGATAActcgagTTATCTG  CCGCCAGTACGTAG-3' |
